# Parenting Styles and Child’s Well-Being: The Mediating Role of the Perceived Parental Stress

**DOI:** 10.5964/ejop.v16i3.2013

**Published:** 2020-08-31

**Authors:** Elisa Delvecchio, Alessandro Germani, Veronica Raspa, Adriana Lis, Claudia Mazzeschi

**Affiliations:** a Department of Philosophy, Social Sciences and Education, University of Perugia, Perugia, Italy; b Department of Developmental Psychology and Socialization, University of Padova, Padova, Italy; University of Belgrade, Belgrade, Serbia

**Keywords:** authoritarian parenting style, authoritative parenting style, parenting styles, parenting stress, well-being

## Abstract

In the last decades, consensus from laymen, scholars, and policy-makers has emphasized the role of child-parent relationships to promote child’s development and positive well-being. Parenting style was claimed as one of the crucial factors for the child’s positive adjustment. The main aim of the present study was to investigate the relationships between authoritative and authoritarian parenting styles and child’s difficulties. The mediational role of parent’s perception of a difficult child on the above mentioned relation was taken into account. The study was carried out on a sample of 459 couples including mothers (n = 459) and fathers (n = 459) of children aged 2 to 10 years old who filled in the Parenting Styles & Dimensions Questionnaire short version, the Strengths and Difficulties Questionnaire, and the Parenting Stress Index-short form. Main findings indicated that authoritative style was associated with less child’s maladjustment, while the authoritarian one showed the opposite association. These relationships were partially mediated by the perception of a difficult child, which partially explained the link between parenting style and child’s problems. Above and beyond the role of parent’s perception as a difficult child, parenting styles had an important effect on child’s difficulties. Future studies should replicate these results with other samples, use the spouse version of the parenting styles, control the effect of socio-economic status and other variables related to family functioning, as well as to consider the child’s perception regarding parents’ parenting style.

Parenting is a complex construct that involves several and multifaceted variables (Bornstein, 2002). It was defined as a normal variation in the parents' attempts of controlling their children and making them socialize (Baumrind, 1967). Parenting includes many specific attitudes which work individually and cumulatively to influence the child's behavior. Schaefer (1959, 1965) proposed a circumplex parenting model using three dimensions of acceptance versus rejection, psychological autonomy versus psychological control, and firm behavioral control versus lax behavioral control. On these dimensions Baumrind (Baumrind 1966, 1968; Baumrind & Thompson, 2002) proposed three parental prototypes: authoritative, authoritarian and permissive, to describe models of parental control and child socialization. For the purposes of the current paper, authoritative and authoritarian styles were taken into account.

Authoritative parents tend to show high acceptance and behavioral control, low psychological control, high responsiveness and warmth to their children. They are conceptualized as affectionate, encouraging, and controlling in a way that promotes child autonomy (Baumrind, 1966, 2013a; Baumrind, Larzelere, & Owens, 2010). They direct the child in a rational situation-based way in which both autonomy and conformity are important, they clearly set rules and use reasoning to enforce them for encouraging open communication, supporting children’s independence, and expressing love and affection (Maccoby & Martin, 1983). Authoritative style was related to positive child outcomes such as self-reliance (Baumrind, 1968), social responsibility (Baumrind, 1971), and adjustment (Baumrind et al., 2010; Braza et al., 2015; García & Gracia, 2009; Luyckx et al., 2011). A large number of studies indicated that children of authoritative parents are shown as being cooperative, friendly, emotionally stable, and with good academic performance (García & Gracia, 2009; Maccoby, 1992; Steinberg, Elmen, & Mounts, 1989).

Authoritarian parents combine high control with low levels of warmth involvement, support, and emotional commitment to their child; they are rejecting, highly demanding, strongly commanding, psychologically and domineeringly controlling (Baumrind, 2013b; Baumrind et al., 2010). They are often punitive and forceful in order to adhere to an absolute standard of behavior (Baumrind, 2012), leaving very little space to the child’s decision making or rationales (Baumrind, 1991). Parents believe that the child should do what they say, as a consequence child’s behaviors and attitudes are guided, formed and based on a certain standard of conduct. This parenting style was related to less optimal child outcomes, including lower self-efficacy (Baumrind et al., 2010), more externalizing and internalizing problems (Braza et al., 2015; Maccoby & Martin, 1983; Rinaldi & Howe, 2012), and rebellion (Baumrind, 1968).

Authoritative parenting style was defined as the optimal parenting style (e.g., Baumrind, 1966, 2013a; García & Gracia, 2009; Luyckx et al., 2011; Maccoby & Martin, 1983) whereas the authoritarian style was proved to be the most negative form of parenting (Baumrind & Black, 1967; Luyckx et al., 2011; Olivari, Tagliabue, & Confalonieri, 2013). Research in the last decades supported Baumrind’s prediction about authoritative parents (Bornstein & Lamb, 2015; Larzelere, Morris, & Harrist, 2013) and the adverse effects of authoritarian parenting.

In order to assess parenting styles, researchers mainly used questionnaires (e.g., Steinberg, Mounts, Lamborn, & Dornbusch, 1991). One of the most known tools is the Parenting Practices Questionnaire (PPQ; Robinson, Mandleco, Olsen, & Hart, 1995). This 62-item self-report measure aimed at identifying continuous scales of authoritative, authoritarian and permissive parenting styles (Robinson et al., 1995), although greater importance was given to authoritative and authoritarian styles. Several variations of this measure have been developed in recent years (e.g., Coolahan, McWayne, Fantuzzo, & Grim, 2002; Robinson, Mandleco, Olsen, & Hart, 2001; Robinson et al., 1995; Russell, Londhe, & Britner, 2013; Wu et al., 2002). Among them, the most used version is the Parenting Styles and Dimensions Questionnaire - short form (PSDQ; Robinson et al., 2001), an abbreviated self-report measure of the PPQ in which each parent independently reports his/her own parenting style and his/her spouse’s parenting style. The PSDQ measures continuous scales of authoritative, authoritarian, and permissive parenting styles, although only some of the original items and dimensions from the PPQ were retained.

PSDQ was widely used in large samples (Padilla-Walker & Coyne, 2011; Topham et al., 2011; Williams et al., 2009) and in different cultures (Kern & Jonyniene, 2012; Önder & Gülay, 2009; Porter at al., 2005). PSDQ psychometric characteristics were reviewed by Olivari et al. (2013). They showed that PSDQ was most frequently used (83.02%) among school-age or pre-school children (Olivari et al., 2013). Little attention was given to the comparison of PSDQ among different ages. A need for further studies was claimed after having found mixed results in the conduction of three studies which included parents of children from nursery to primary school (Olivari et al., 2013). Moreover, none of them were carried out in Italy. Notwithstanding the increasing interest on parenting styles (Olivari et al., 2013), there are still many issues that need to be addressed.

The present study had two main goals. The first aim was to contribute to the investigation of the authoritative and authoritarian parenting styles by using the PSDQ in a large sample of Italian parents of children aged 2-11 years old. The second purpose was to focus and expand the investigation on the relationship between the authoritative and authoritarian parenting styles and child's adaptation. The possible mediator role of parents’ perception about having a difficult child was also examined. Mother and father’s contributions were taken into account separately.

## Issues About the PSDQ

Previous research comparing parenting styles in different cultures showed that the original assumption that authoritative style would be the “best protective” style of parenting, is not always true. For instance, in African American families, authoritarian parenting, if associated with physical punishment, did not increase the level of behavior problems, suggesting that the pattern of findings reported by Baumrind is culture-specific (Baumrind, 1972). A large body of evidence supports the importance of extending investigation on parenting and its related contribution to child development, as well as to collect data in countries that can be culturally different concerning parenting practices (Bornstein et al., 1998; Russell, Hart, Robinson, & Olsen, 2003).

In Italy, the parent-child relationship is characterized by basic emotional bonding and support from parents. Moreover, although parents encourage their children to be autonomous and independent (Casiglia, Lo Coco, & Zappulla, 1998; Confalonieri, 2010; Laudani, Guzzo, Cascio, Pace, & Cacioppo, 2014; Scabini & Cigoli, 2000), attention is also paid to the rights of children in a two-way parent-child relationship (Scabini, Marta, & Lanz, 2006; Yeh & Bedford, 2004). However, a parental change involving fathers (Bacchini, Galiani, Guerriera, & Sbandi, 2003) was observed to occur among Italian culture in the last 20 years (Confalonieri, 2010). From a family where the parents’ roles were differentiated, with the mother being in charge of caring for the child whereas the father providing rules and transmitting values (Confalonieri et al., 2010), there was a shift toward a family where both mother and father identify their main function as to ensuring care to their children. However, just one Italian study (Confalonieri, Giuliani, & Tagliabue, 2009) was run on parenting styles of parents with children aged 3 to 10.

Attempts to compare mothers’ and fathers’ approach to parenting styles yielded inconclusive findings. Baumrind (1991, 2013a) focused on both parents rather than on single mothers or fathers, but she didn’t explore why parents adopted these strategies. Studying parents of Australian and US pre-schoolers, Russell and colleagues (2003) found that mothers were more likely to identify with the authoritative style of parenting, whereas fathers were more likely to describe themselves as either authoritarian or permissive. Based on these results, we expected the highest average score for authoritative style in mothers and on the contrary the highest average score for authoritarian style for fathers (Hypothesis 2). However, because of the shift occurred in Italy where both parents identify their main function as to ensuring warm care to their child, both mothers and fathers were expected to report higher scores in authoritative than authoritarian style (Hypothesis 1).

Another limitation in the existing literature is the investigation of possible differences in parenting styles related to the child’s gender. Few papers were devoted to this issue. Russell et al. (2003) found that more authoritative parenting was reported by parents of girls rather than by parents of boys, while more authoritarian parenting was found in parents of boys rather than of girls. The same results were hypothesized for the present study (Hypothesis 3). Finally, although many papers were focused on children of different ages, few studies compared possible variation of parental styles with the child’s developmental changes with regard to age. Bornstein and Lamb (2015) reviewed and stressed the importance of different roles played by parents in their involvement with children in pre-school and school years, respectively taking into account the different goals of development the child needs to reach in these two important age groups. Thus, the comparison of parenting styles in these two periods needs more investigation and no specific hypothesis was given (Hypothesis 4).

## PSDQ and Related Variables

A considerable research body examined how variations in parental styles are related to a wide range of child outcomes (Baumrind et al., 2010; Braza et al., 2015; García & Gracia, 2009; Luyckx et al., 2011). Relationship between parenting styles and child’s behavioral problems were examined. For example, as said before, authoritative style was related to psychological adjustment (Baumrind et al., 2010), whereas the authoritarian one was positively related to externalizing problems (Braza et al., 2015; Maccoby & Martin, 1983; Rinaldi & Howe, 2012). Researchers described the relationship between parenting styles and some negative outcomes concerning academic adjustment, success, moral development, emotion regulation and social competence (Davidov & Grusec, 2006; Denham et al., 2000; Laible, 2004; Spera, 2005). However, Darling and Steinberg (1993), Bornstein and Lamb (2015), and Russell et al. (2003) pointed out that the influence of parenting style on behavioral outcomes across ethnic groups and cultures was not adequately studied, despite research findings had suggested that differences exist.

Previous research reported greater parenting stress regarding parents with an authoritarian parenting style (Deater-Deckard & Scarr, 1996; McBride & Mills, 1993), whereas other studies showed no differences in parenting distress among authoritarian and authoritative styles (Woolfson & Grant, 2006). Parenting stress is a universal experience for parents in all sociodemographic groups and contexts (Crnic & Low, 2002). Parents who experience moderate amounts of parenting stress may engage in lesser optimal parenting (Bonds, Gondoli, Sturge-Apple, & Salem, 2002; Seginer, Vermulst, & Gerris, 2002) and the children of stressed parents may be adversely affected, though indirectly, through parenting behaviors (Magill-Evans & Harrison, 2001; Putnick et al., 2008, 2010; Seginer et al., 2002). Within parenting stress, Abidin (1995) and Deater‐Deckard (1998) identified a specific dimension: stress that can be attributed to the parent’s perception of the child as being difficult. More specifically, it deals with the child’s behavioral characteristics that make him or her easy or difficult to manage, due to either temperament and/or noncompliant, defiant, or demanding behavior. However, no previous studies on the link between parenting styles and parent’s perception of a difficult child were found.

Based on the above-mentioned findings, we hypothesized that the authoritative style was negatively related to behavioral problems in children, whereas the authoritarian one was positively related to them (Hypothesis 5). Moreover, in this study, we explored how a perception of the child as being difficult would mediate the relationship between authoritative and authoritarian parenting style and the child’s behavior difficulties. Since a negative relation between authoritative style and the perception of a difficult child, as well as a positive relation between the authoritarian style and the perception of a difficult child were expected (Hypothesis 6), negative and positive indirect effects of parenting styles on child’s behavioral difficulties were hypothesized respectively (Hypothesis 7).

Thus, to summarize, the current study is aimed to investigate in mothers and fathers respectively (a) levels of authoritative *versus* authoritarian parenting style and the role of parent’s gender; (b) the role of child’s gender and age on parenting styles and (c) the relation between parenting styles and child’s psychological well-being mediated by parents’ perception of having a difficult child.

## Method

### Participants

Participants were 459 Caucasian parent couples (*n* = 459 fathers, *M*_age_ = 37.13, *SD* = 5.39; and *n* = 459 mothers, *M*_age_ = 35.08, *SD* = 4.95) living in urban and suburban areas of Central Italy. They were married or living together heterosexual parents of children from infancy to childhood (2-11 years old). Considering the large life-span included, parents were assessed considering child’s developmental stage: (a) pre-school children (from 2 to 5 years old) and (b) school aged children (from 6 to 11 years old). 239 (*n* = 129 boys (54.0%), *M*_age_ = 3.21, *SD* = 1.39) were parents of pre-school age children; 220 (*n* = 108 boys (49.1%); *M*_age_ = 7.91, *SD* = 1.33) were parents of school age children. More than half (58.5%) were parents at their first parenthood experience. Parents’ socio-economic level measured by SES (Hollingshead, 1975), was primarily middle to upper for 90% of families. Only 7% had a low socio-economic status and only 3% reported a very high level.

### Procedure

All procedures were conducted in compliance with the ethical standards for research outlined in the 1964 Helsinki declaration. Both parents gave written informed consent to participate in the study. Confidentiality was assured in all phases of the study using a numerical code instead of participants’ names. Families were recruited through day-cares, nurseries and schools. Parents who participated in the study were given at their children’s school - with the teachers’ help - a booklet containing the tools that they needed to fill in at home and bring back to school. No incentives were awarded and voluntary participation was encouraged. Families of the final sample met the following criteria: (a) both mothers and fathers agreed to participate, (b) all participants completed the entire assessment phase (c) parents and children did not meet criteria for psychiatric diagnosis and were not under psychological treatment. 5% of the initial participants did not meet all the criteria.

### Measures

#### Parenting Styles & Dimensions Questionnaire- Short Version (PSDQ)

The PSDQ-short version (Robinson et al., 2001) is a 32 self-report questionnaire measuring continuous dimensions of parenting styles using *authoritative* (15 items), *authoritarian* (12 items) and *permissive* (5 items) scales (Russell et al., 2013). It comprehends a self and spouse version (i.e., self-evaluation of her/his-own parenting style and evaluation of his/her spouse’s parenting style). With regard to the current study, the authoritarian and authoritative scales of the self-version were considered. Mothers and fathers independently rated themselves by assessing how often they exhibited parenting behaviors as described in each item by using a five-point scale from “*never*” to “*always*” (coded 1 to 5). Higher mean scores indicated a prevalence on that style. Confalonieri, Giuliani, and Tagliabue (2009) adapted the measure with Italian parents. The alpha value related to the authoritarian scale was .70 (.65-.74) for fathers, and .72 (.69-.76) for mothers, whereas referring to the authoritative scale it was .78 (.75-.81) for fathers and .75 (.72-.78) for mothers.

#### Strengths and Difficulties Questionnaire (SDQ)

The SDQ (Goodman, 1999, 2001) is a brief questionnaire of 25 attributes, some positive and some negative, for assessing strengths and difficulties in the psychological adjustment of children and adolescents. Each item uses a 3-point ordinal Likert scale (0 “*not true*”; 1 “*somewhat true*”; 2 “*certainly true*”). In the present paper, the Total Difficulties Score (TDS; 20 items, range 0-40) was used. Higher scores indicated more difficulties^i^. Cronbach’s coefficients for SDQ TDS were .79 (.76-.81) for fathers and .78 (.74-.80) for mothers.

#### The Difficult Child Subscale (PSI_DC) of the Parenting Stress Index-Short Form (PSI-SF)

The PSI-SF (Abidin, 1995) is a 36-item measure of overall levels of stress experienced by parents. The current study is focused on the Difficult Child Scale which includes 12 items scored on a 5-point Likert scale from 1 (*Strongly agree*) to 5 (*Strongly disagree*). It measures the parent’s perception of his/her child’s self-regulatory abilities. The higher the score is, the higher the perceived difficulties will be. The measure was validated in several countries, among which Italy (Guarino, Di Blasio, D’Alessio, Camisasca, & Serantoni, 2008). Cronbach’s alpha coefficients for the present study were .88 (.86-.89) for fathers and .86 (.84-.88) for mothers.

### Data Analysis

Firstly, in order to test Hypotheses 1-4, descriptive statistics and a MANOVA were performed with parental role, child’s gender, and age-group as factors, and the authoritative and authoritarian parenting styles as dependent variables. Effect size was measured by using partial eta-squared, in which small, medium, and large effects were .0099, .0588, and .1379, respectively (Cohen, 1988, p. 283). Second, before testing the mediational model, Pearson’s correlations were run to explore the associations between the selected variables. Effect sizes were interpreted according to Cohen (1992), with correlation coefficient of .10, .30, and .50 representing low, medium and high effect sizes, respectively. Third, the mediational models split for mothers and fathers were carried out. Specifically, in order to test the total effects of parenting styles on children’s maladjustment (Hypothesis 5), simple regression analyses were used. Then, to test their effects on difficult child variable (i.e., the mediator; Hypothesis 6), simple regressions were analyzed. Finally, in order to assess direct and indirect effects of parental styles on child’s behavior problems mediated by the perception of a difficult child, SPSS Process Macro (Model 4) developed by Preacher and Hayes (2008) was used (Hypothesis 7). This allowed the authors to observe the change of the total effects and the influence of the mediator (i.e., the mediated effect). Bootstrapping method (Preacher & Hayes, 2008) was applied to all the mediation models. Specifically, 5000 bootstrap samples were used from the full data and 95% confidence interval was utilized to determine the significance of the mediating effect. Significant mediating effect was identified if the confidence interval excluded 0. In order to calculate the ratio of the indirect effect to the total effect, proportion mediated (PM = indirect effect/total effect) was calculated. Regression and moderation analysis were carried out separately for mothers and fathers. Analyses were carried out using SPSS (Version 18.0) and Process Macro for SPSS.

## Results

### The Effect of Parental Role, Child’s Age, and Age-Group on Authoritative and Authoritarian Parenting Styles

Means and standard deviations of PSDQ subscales for mothers and fathers, and according to child’s gender and age-group are shown in Table 1.

**Table 1:** Descriptive Statistics of PSDQ Variables

PSDQ	Mothers		Fathers		Preschool		School		Boys		Girls	
	*n* = 459		*n* = 459		*n* = 239		*n* = 220		*n* = 237		*n* = 222	
	*M*	*SD*	*M*	*SD*	*M*	*SD*	*M*	*SD*	*M*	*SD*	*M*	*SD*
Authoritative	3.88	0.51	4.11	0.39	3.98	0.46	4.01	0.47	4.02	0.46	3.97	0.47
Authoritarian	2.04	0.45	2.15	0.45	2.07	0.46	2.12	0.44	2.11	0.47	2.09	0.43

Results of MANOVA indicated that there was a significant main effect of parental role (*Wilks’ Lambda* = .914, *F*(2, 909) = 43.00, *p* < .001, η^2^ = .086) on dependent variables (parenting styles), but there were not significant main effects of child’s gender (*Wilks’ Lambda* = .996, *F*(2, 909) = 2.04, *p* = .131, η^2^ = .004) and age-group (*Wilks’ Lambda* = .994, *F*(2, 909) = 2.62, *p* = .074, η^2^ = .006) on parenting styles. ANOVA indicated that there was a significant effect of parental role both for authoritative (*F* = 57.96, *p* < .001, η^2^ = .060) and authoritarian (*F* = 2.83, *p* < .001, η^2^ = .015) styles. As shown in Table 1, fathers referred both a higher authoritative and authoritarian style than mothers, with a medium and small effect size, respectively.

### Correlations Between Selected Variables by Parental Role

Pearsons’ correlations are reported in Table 2. Authoritative and authoritarian styles were significantly and negatively correlated to each other with small effect size, independently of parental role. Moreover, on one hand, authoritative style was significantly and negatively correlated to the perception of a difficult child and child’s total difficulties with small effect sizes, irrespective of parental role. On the other hand, authoritarian style was significantly and positively associated to parents’ perception of a difficult child and child’s total difficulties, with small effect size, both in mothers and fathers. Finally, the perception of having a difficult child and child’s total difficulties were significantly and positively associated both in fathers and mothers, with medium and nearly large effect size, respectively.

**Table 2:** Pearson’s Correlations Between the Selected Variables, Split for Parental Role

Variable	1	2	3	4
1. Authoritative style		-.19*	-.18*	-.13*
2. Authoritarian style	-.19*		.29*	.22*
3. Difficult child	-.13*	.18*		.49*
4. Child’s total difficulties	-.15*	.22*	.35*	

*Note.* Coefficients above the diagonal referred to mothers, coefficients under the diagonal referred to fathers.

*p < .01.

### Parenting Styles, Parenting Stress and Child’s Psychological Well-Being

**Figure 1:**
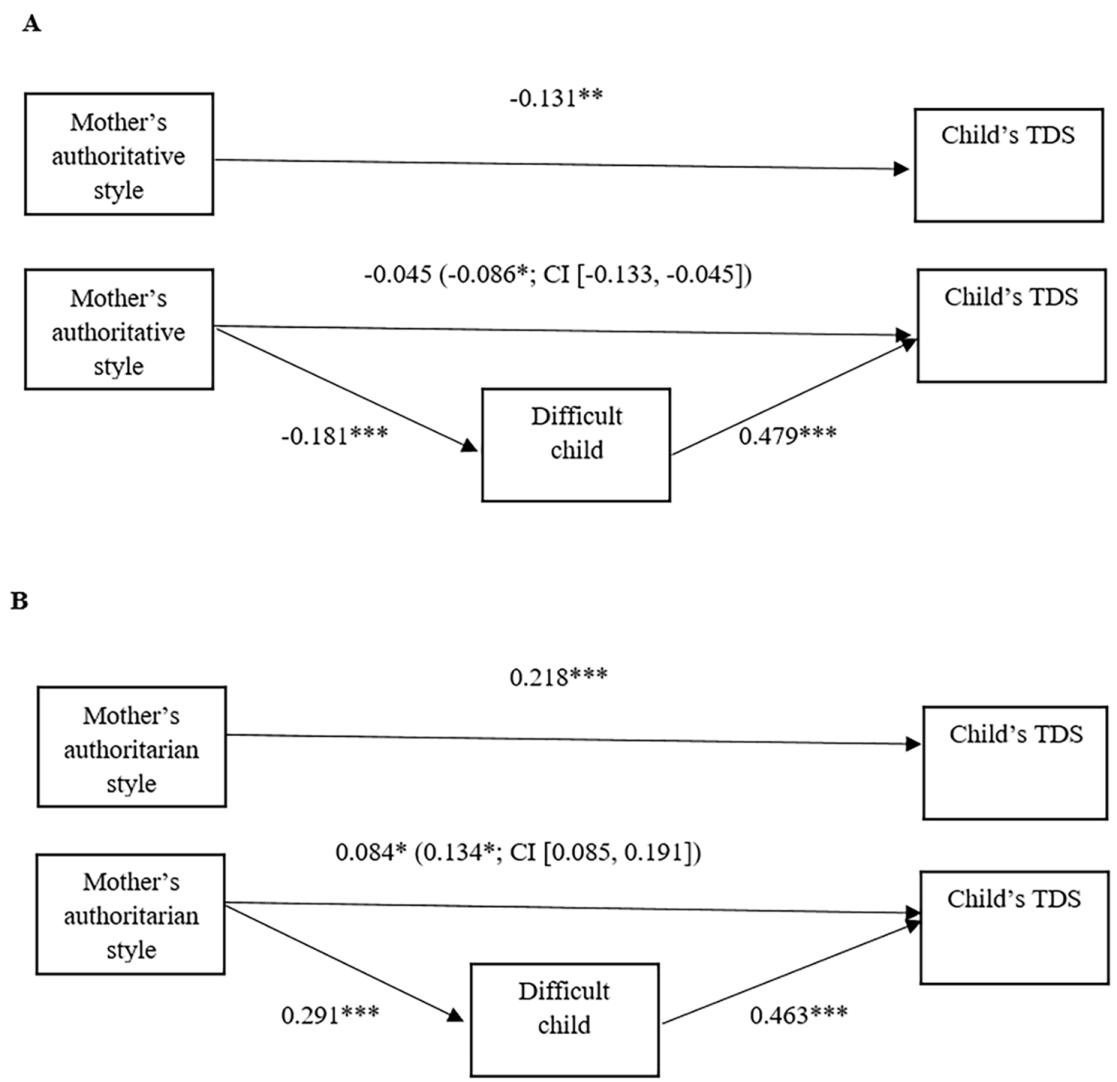
Regression and mediational model for mothers. Total effects and mediational models examining the direct and indirect effects of authoritative (A) and authoritarian (B) parenting styles of mothers on child’s TDS, mediated by the perception of having a difficult child. *Note*. Slope coefficients are standardized; numbers in parenthesis represent significant indirect effects mediated by Difficult Child (5.000 bootstrap samples, 95% confidence interval). *p < .05. **p < .01. ***p < .001.

**Figure 2:**
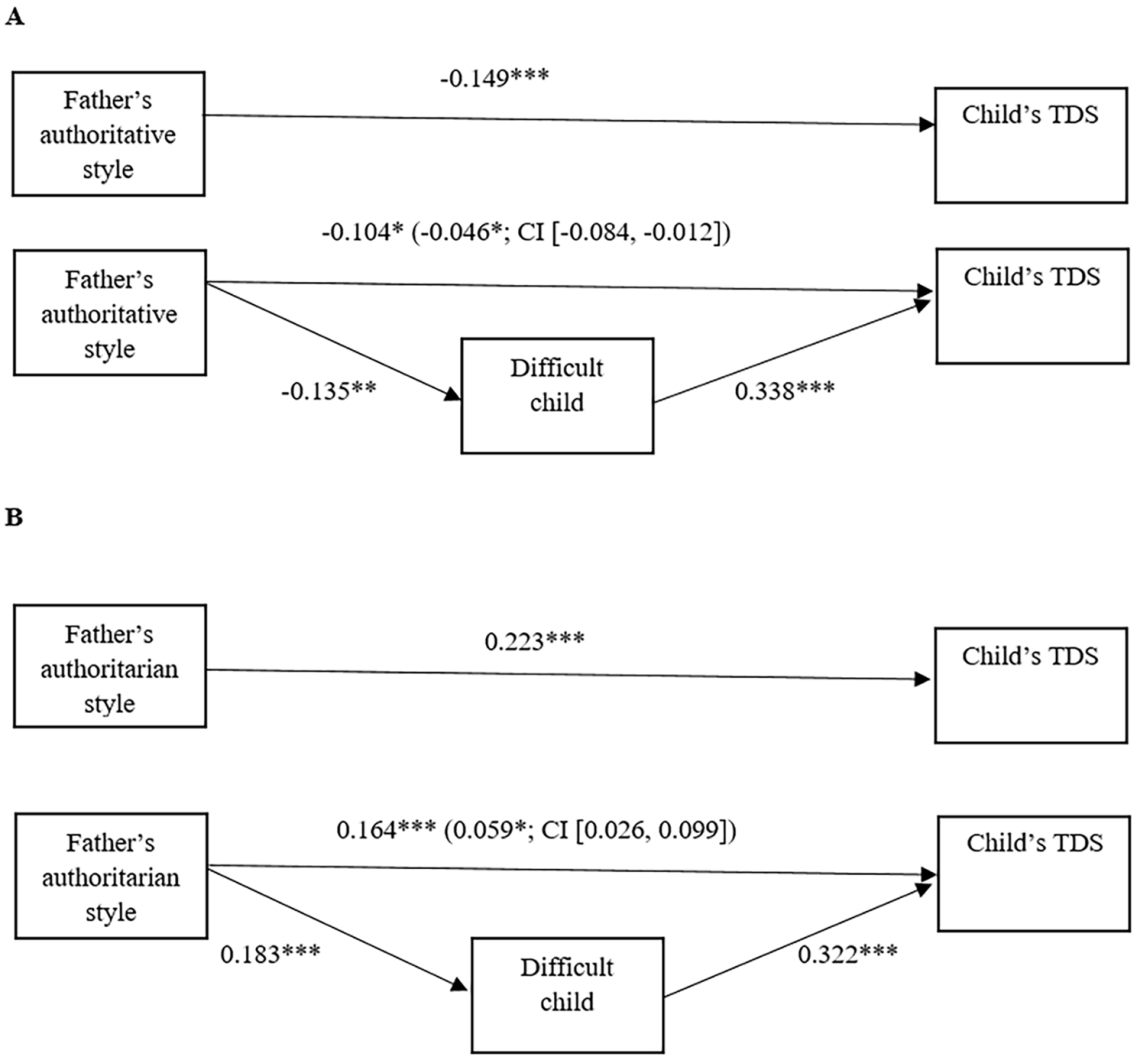
Regression and mediational model for fathers. Total effects and mediational models examining the direct and indirect effects of authoritative (A) and authoritarian (B) parenting styles of fathers on child’s TDS, mediated by difficult child variable. *Note*. Slope coefficients are standardized; numbers in parenthesis represent significant indirect effects mediated by Difficult Child (5.000 bootstrap samples, 95% confidence interval). *p < .05. **p < .01. ***p < .001.

Since results for mothers and fathers showed similar paths, they are described together, although the statistics are illustrated separately in Figure 1 and Figure 2 respectively.

Referring to authoritative style (Figure 1A and 2A): i. Total effect. The increase in authoritative style significantly predicted a decrease in TDS; ii. Variation in mediator. The rise in authoritative style predicted a decrease in the score of the perception of a difficult child; iii. Mediation model. The increase in the difficult child variable predicted a rise in the child’s TDS. Moreover, authoritative style showed both negative direct (except for mothers) and indirect effect on the child’s total difficulties as mediated through the perception of a difficult child variable. More specifically, the proportion mediated by the difficult child variable was .65 (65%) for mothers and .31 (31%) for fathers.

Regarding the authoritarian style (Figure 1B and 2B): i. Total effect. There was a positive relation between authoritarian style and TDS score; ii. Variation in mediator. Moreover, the rise in authoritarian style predicted an increase of the difficult child variable; iii. Mediation model. The perception of a difficult child had a positive relation with the child’s TDS. Moreover, the authoritarian style showed both positive direct and indirect effects on the child’s TDS. The proportion mediated by difficult child variable was .61 (61%) for mothers and .26 (26%) for fathers.

## Discussion

The first aim of this paper was to investigate the authoritative and authoritarian parenting styles using the PSDQ in a large sample of Italian parents of children aged 2-11 years old. Bornstein and Lamb (2015) stressed how greatly research enriched understanding of socialization processes with family system scholars, documenting the processes by which parents and children shape one another’s behavior. The authors highlighted the developmental processes by which authoritative parents facilitate their children’s socialization (Bornstein & Lamb, 2015). Jacobson and Crockett (2000) and Smetana (2008) reported that parental involvement plays an important role in the development of both social and cognitive competences in children, whereas a lack of it is associated with the risk of delinquent behavior. Bornstein and Lamb (2015) according to these results, suggested to consider both the children’s impact on the parents’ child rearing beliefs and the parental role in giving care to the child. They stressed that this assertion needs to be explored empirically in a wide variety of cultural and subcultural settings.

The interest in Italian parents originated from many different issues. First of all, the importance to extend investigation of parenting styles in order to advance the understanding of factors shaping parenting as well as processes that influence child development (Russell, 2003; Wu et al., 2002). Italy is a country where parenting is characterized by intertwining emotional bonding and supportive parent-child relationship with encouragement for the child’s autonomy. Moreover, both mothers and fathers are called to ensure the care for the child. The complexity of the present parenting “environment” confirmed the hypothesis that both mothers and fathers reported higher score on authoritative than on authoritarian parenting style. However, for both styles, fathers scored higher than mothers. Following the general agreement in literature as to the positive effect of authoritative versus authoritarian styles, Italian parents seem to offer their children a healthy environment characterized by warmth, affection, open communication and supportive proximity (Maccoby & Martin, 1983). Although gender and age-group differences were expected, mothers and fathers appeared to be homogenous in their score of authoritative and authoritarian parenting styles.

A further aim of this study was to focus and expand the investigation of the relationship regarding the authoritative and authoritarian parenting styles with the child's adaptation. More specifically, to investigate the possible relationship between these two parenting styles in mothers and fathers and their perception of the child’s well-being. Stressful perception of a difficult child was also examined as a mediator. The significant total effects of parenting styles on the child’s behavioral problems confirmed this well-established relation. More precisely, the authoritative parenting style had a positive influence in diminishing the parent’s perception on the child’s maladjustment, and the reverse for the authoritarian style. These results were regardless of parent’s gender. In line with literature, these results confirmed that the authoritative style is the optimal parenting style while the authoritarian style is a less desirable form of parenting (Baumrind, 1966, 2013a; Bornstein & Lamb, 2015; Larzelere et al., 2013). The current study allowed confirming this trend also for Italian mothers and fathers. Moreover, being the fathers more authoritative than authoritarian, just like mothers, they appeared to contribute in their protecting role against child’s maladjustment.

The potential role of parents’ perception of a difficult child in mediating the aforementioned relation was investigated. As expected, the higher the authoritative style was, the lower the parents’ perception of a difficult child and referred problems. On the contrary, parents who reported higher score in the authoritarian style, referred that their children were particularly difficult, and they reported higher difficulties in their children. Both mothers and fathers showed the same pattern. In other words, both parents were significantly less stressed by their perception of the child as difficult when their style was authoritative and the reverse when their style was authoritarian. Moreover, the higher the parental distress related to their perception of a difficult child, the more problems in children arose. Therefore, being authoritative has indirect protective effect on child’s behavioral problems, because it is related to less perception of a difficult child. On the contrary, being authoritarian increases the child’s problems, also because parents consider more difficult their children. This is true for mothers and fathers, although, mothers were significantly more influenced by the perception of their child as difficult. In other words, this component of parenting stress seemed to explain more variance in child’s total difficulties in mothers than in fathers. A possible explanation may be that mothers, as primary caretakers, have to deal more closely with everyday life and routine with the child than fathers, even if the latter in the last few years have been more involved in family life. This makes mothers more sensitive to stress linked with child care. Finally, it is important to highlight that the present study allowed to measure the relation between parental styles and child’s behavioral problems taking into account and controlling for the role of a relevant aspect of parenting stress (i.e., parents’ perception of a difficult child). In other words, the current work emphasized the link between parenting styles and child’s behavioral problems above and beyond the parents’ evaluation of the child as difficult.

This study has some limitations. Firstly, this study used a specific measure of parenting with respect to other versions quoted in literature. Moreover, although in the current study the self-report version of the PSDQ was administered to mother and father of each child, the spouse version of the PSDQ was not considered. Since the self-report and the spouse version could offer different results, future studies are encouraged to use both versions to delve into these issues. Furthermore, the categories selected (i.e., pre-school versus school age children) may be too ample to make the differences of parenting styles linked to child’s age emerge. Greater and more detailed samples need to be taken into consideration in order to make more generalizable and conclusive results. Hoff, Laursen, and Tardif (2002) described the strong association between parenting and socioeconomic status. Although the current paper assessed parents’ SES, it did not consider such variable for data analysis. Neither the role of parental burden was taken into account, although it may play a role on the selected variables (e.g., Oyserman, Bybee, Mowbray, & Kahng, 2004). Future studies should deepen into those topics, due to their meaningful potential role in explaining such complex relationships. Furthermore, the current study used only parents’ perceptions. Further studies could be carried out to analyze child’s perception regarding parents’ parenting style (according to child’s developmental stage).

Notwithstanding, the present study contributes to the cross-cultural literature on parenting styles in pre-school and school children, as well as to the association with the child’s difficulties. The results of the mediational models highlight that stress connected to the perception of the child as difficult, is one of the mechanisms explaining how parenting styles are related to psychological difficulties in Italian pre-school and school age children.
